# A single pleiotropic locus influences the rate of hybridization between two sibling species of *Lygaeus* bugs

**DOI:** 10.1002/ece3.6853

**Published:** 2020-09-29

**Authors:** Vicki L. Balfour, Daniella Black, David M. Shuker

**Affiliations:** ^1^ School of Biology University of St Andrews St Andrews UK

**Keywords:** hetero‐specific, hybridization, *Lygaeus equestris*, *Lygaeus simulans*, mating failure, mating rates

## Abstract

The evolution of reproductive isolation lies at the heart of understanding the process of speciation. Of particular interest is the relationship between pre‐ and postzygotic reproductive isolation, and the genetic architecture of traits that contribute to one or both forms of reproductive isolation. The sibling species of seed bug *Lygaeus equestris* and *L*. *simulans* show a classic pattern of asymmetric prezygotic reproductive isolation, with female *L. equestris* hybridizing with male *L. simulans*, but with no hybridization in the reciprocal direction. We have recently described a mutant pale color form of *L. simulans*, that inherits as a single Mendelian locus and is pleiotropic for a number of other life history and behavioral traits. Here, we tested whether this locus also influences pre‐ and postzygotic reproductive isolation. Two sets of experimental crosses revealed that behavioral isolation varied with mutant versus wild‐type phenotype for male *L*. *simulans*, with the pale form less successful at mating with female *L*. *equestris*. In terms of trying to assess postzygotic isolation, levels of hybrid offspring production were uniformly low across the experiments. However, we did obtain, for the first time, hybrid offspring from a pairing between a female *L. simulans* and a male *L. equestris*. In this instance, the female was of the pale mutant genotype. Together with evidence for heterozygote advantage in terms of nymph survival, we consider our results in terms of possible mechanisms of reproductive isolation between this species pair, the role of the pale mutation, and the possible genetic architectures underlying the mutation, from a single gene to a supergene.

## Graphical abstract

A pale mutant *Lygaeus simulans* female copulating with a wild‐type male *L. simulans*. Here, we show that the pale mutation in *L. simulans* influences hybridization with its sister species *L. equestris*. In particular, we found that pale males were less successful at hybridizing than wild‐types. This work confirms the highly pleiotropic nature of the pale locus. Photo credit: Vicki Balfour.

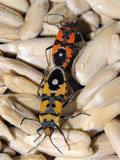

## 1 INTRODUCTION

The evolution of reproductive isolation is central to the process of speciation (Coyne & Orr, 2004; Nosil, 2012; Butlin & the Marie Curie SPECIATION Network, 2012; but see also Harvey et al., 2019). Increasing evidence has amassed concerning the various forms of pre‐ and postzygotic isolation that have evolved between populations in allopatry through to sympatry (Coyne & Orr, 2004; Nosil, 2012), and some general patterns have emerged. For instance, processes of prezygotic reproductive isolation, including mechanisms of behavioral isolation such as habitat or mate choice, typically evolve before postzygotic isolation (Coyne & Orr, 1989, 1997). Reproductive isolation between populations or recently diverged species is also often asymmetric, with gene flow possible in one direction of cross, but not in the other (Coyne & Orr, 1989, 1997; Yukilevich, 2012). In terms of postzygotic isolation, the clearest pattern of all perhaps is Haldane's Rule. Haldane's Rule states that if hybrid sterility or hybrid inviability is not complete in both sexes, then it will be most strongly (or only) expressed in hybrids of the heterogametic sex (Davis et al., 2015; Haldane, 1922; Orr, 1997; Presgraves, 2008; Presgraves & Orr, 1998).

The focus of much contemporary work on reproductive isolation and speciation has been the underlying genetic changes that either lead to, or are otherwise associated with, divergence between populations (Butlin & the Marie Curie SPECIATION Network, 2012; Nosil & Schluter, 2011). This has included the search for genes directly shaping pre‐ or postzygotic reproductive isolation, and understanding broader genomic changes linked to speciation and its consequences. This search has a long history. For instance, right back at the beginning of modern experimental genetics, Sturtevant (1915) showed that female *Drosophila melanogaster* with the *yellow* mutation were more likely to hybridize with male *D*. *simulans* than wild‐type females (see also Sturtevant, 1920; Provine, 1991 provides a historical review of Sturtevant's work on hybridization in *Drosophila*). Various species of *Drosophila* have since played a major role in understanding hybridization and the genetic basis of reproductive isolation. This body of work has encompassed mutations that first appeared de novo in laboratory stocks (such as *yellow*), induced mutations via different forms of mutagenesis or deletion mapping, as well as screens of standing genetic variation, including genomic screens (Castillo & Barbash, 2017; Coyne & Allen Orr, 1998; Coyne & Orr, 1997, 2004; Orr, 2005; Phadnis et al., 2015).

The use of spontaneously occurring mutants in the study of reproductive isolation is therefore well‐established. Here, we consider the role of a single spontaneous mutation on pre‐ and postzygotic reproductive isolation between two species of seed bug, *Lygaeus equestris* and *L*. *simulans* (Hemiptera, Heteroptera: super‐family Lygaeidae). The two sibling species have only been relatively recently distinguished (Deckert, 1985), and they overlap in distribution in central‐western Europe (Evans et al., 2015). To date, to the best of our knowledge, no hybrids have been found in the field (Maschler, 2002). We have previously shown that female *L. equestris* can mate and hybridize with male *L. simulans*, but not the other way around, an example of the asymmetric reproductive isolation described above (Evans et al., 2015). We have also recently isolated and described a pale mutant color morph of *L. simulans* (Balfour et al., 2018). Although no evidence for this pale mutant exists in the wild, a potentially similar “gray” mutant was recorded in the laboratory by Sillén‐Tullberg (1985) in *L. equestris*. The pale mutant is a recessive mutation that inherits as a single Mendelian locus, and this color locus appears to be pleiotropic with other life history and behavioral traits (Balfour et al., 2018; Black, Potapova, Balfour and Shuker, unpublished data). The mutation also does not appear to be sex‐linked nor sex‐limited and is presumably autosomal. Because of this rather widespread pleiotropy, here we asked whether the mutant also influenced the nature and extent of hybridization between these two species and whether having a pale *L. simulans* allele instead of a wild‐type allele at this locus influenced the fitness of the hybrid offspring. For instance, the pleiotropic effect of the mutation could be more pronounced in a hybrid background due to genomic incompatibilities between the species.

We performed two sets of experimental con‐ and hetero‐specific crosses between *L. equestris* and both color morphs of *L. simulans*. We aimed to investigate not only differences in hybridization rates, but also differences in mating rates, egg production, nymph production, and the prevalence of mating failure (the absence of offspring production: Greenway et al., 2015). We hypothesized that different combinations of the *L. simulans* color allele with the *L. equestris* genome would cause fitness differences in hybrids, similar to those seen in homozygote and heterozygote *L. simulans* (Balfour et al., 2018). Therefore, we predicted that there would be variation in fitness (egg to nymph viability) between hybrids from crosses of pale *L. simulans* with *L. equestris* and crosses of wild‐type *L. simulans* with *L. equestris*.

## 2 MATERIALS AND METHODS

### 2.1 Insect husbandry

*Lygaeus equestris* were originally collected from Sicily in 1996 and have been reared in continuous lab culture ever since (Shuker et al., 2006). *Lygaeus simulans* were collected in Tuscany, Italy, in 2008 and 2009. The bugs were reared in continuous culture population cages (30 × 15 × 15 cm plastic boxes) supplied with an ad libitum supply of sunflower seeds, two cotton‐bunged water tubes (deionized water; 25 ml), and a piece of cotton wool for habitat. Water tubes were changed once a week. Population cages were kept in the incubator at 29°C and a 22:2 hours light:dark cycle to prevent the onset of reproductive diapause. A minimum of two replicate population cages were kept at any one time. New population boxes were made by transferring 50 bugs of each instar category (i.e., nymphs to adults) from at least two population cages to a new box. This was to reduce inbreeding and enhance gene flow. Pale mutants appeared in the *L*. *simulans* population in 2012 and were isolated into separate population cages in 2013 (see Balfour et al., 2018 for details of the pale mutants).

For the experimental crosses, we collected late instar nymphs from population cages using an aspirator and placed them in nymph boxes (20 × 10 × 8 cm plastic boxes) which contained an ad libitum supply of sunflower seeds, a cotton‐bunged water tube (25 ml), and a piece of cotton wool for habitat. We kept nymphs of each species in separate tubs, and *L. simulans* nymphs of different color morphs were also raised separately.

We checked nymph boxes every 2–3 days for newly eclosed adults. We separated adults by sex, species and color morph into tubs (108 × 82 × 55 mm plastic deli tubs), with a maximum density of 10 bugs per tub. This was to ensure adults used in the experiment were virgins, as female bugs become sexually mature at 7 days old, and males a little earlier. Each tub was again provided with an ad libitum supply of sunflower seeds, a cotton‐bunged water tube (7 ml), and a piece of cotton wool.

### 2.2 Experiment 1

For our first set of experimental crosses, on day 1, we paired up virgin males and females (7–13 days posteclosion) in individual tubs (108 × 82 × 55 mm) with 20–30 sunflower seeds and a cotton‐bunged water tube (7ml). There were nine treatments: EE, EP, EW, PE, PP, PW, WE, WP, WW. The first letter represents the species and genotype of the female, the second letter the male (E = *L. equestris*, P = pale mutant *L. simulans*, W = wild‐type *L. simulans*). The sample sizes were: *N* = 60, 62, 52, 61, 64, 49, 60, 60, and 51 respectively (total *N* = 519 crosses). Following losses during the experiment (such as death or escape of either one or both members of the pair), the final sample sizes ranged from *N* = 46 to 59 (total *N* = 495). Note here that these treatment abbreviations are different to that used in Experiment 2 of Balfour et al. (2018), in which, for example, WP implied a cross between two heterozygous individuals (phenotype = wild type) whose mother had been homozygous for the wild‐type allele, and whose father had been homozygous for the pale allele. In Balfour et al. (2018), only nymphs were counted in the F2 generation, as the F1 nymphs needed to be raised to adulthood so to attain an F2 generation, and nymphs need to be euthanized to be able to accurately count them and avoid sibling cannibalism. Therefore, the results we attained in this paper, of nymph and eggs counts for the F1 generation, should be complimentary to our previous data collected in Balfour et al. (2018).

For the first 2 hr, we scored pairs every 15 min for whether they were engaged in copulation (yes/no) in the typical back‐to‐back copulatory position for these species. Copulation in this species can last anything from as little as 30 min to 24 hr (Kugelberg, 1973; Sillén‐Tullberg, 1981). Though copulations can be less than 30mins, such couplings do not result in successful sperm transfer (Gschwentner & Tadler, 2000). The bugs are highly promiscuous (Burdfield‐Steel & Shuker, 2014) and in the laboratory pairs have been recorded to mate as many as 40 times in their lifetime (Kugelberg, 1973). Additionally, there appears to be no refractory period for females, as females have been observed engaging in copulation <15 min after the termination of copulation, and often with the same male (VB, personal observation). After the initial 2 hr observation period, we transferred tubs to the incubator and pairs were allowed to mate for a further 2 days. During this time, we scored twice daily for whether they were copulating or not (4 checks in total).

On day 3, we separated pairs and euthanized the males by freezing them at −18°C. We left females in their tubs for a further 5 days to lay eggs. Females were then also euthanized. We scored tubs for the presence/absence of eggs, counted any eggs present and discarded any tubs without eggs. We then returned the tubs with eggs to the incubator for a further 7 days. We then froze the tubs at −18°C for a minimum of 24 hr. We then scored tubs for the presence/absence of nymphs and we counted any nymphs present according to color morph.

### 2.3 Experiment 2

We performed a second set of experimental crosses to try and increase the sample size of hybrid offspring (see Results). Therefore, we used only hetero‐specific crosses, resulting in a total of four treatments: EW, WE, PE, and EP (abbreviations as before). The sample sizes were: *N* = 97, 101, 99, and 98, respectively (total *N* = 395 crosses). Again, following experimental losses, the final sample sizes ranged from *N* = 81–95, with total *N* = 360 crosses.

The crosses were undertaken as above, except that pairs were not observed every 15mins for the first two hours of the experiment and pairs were left in the incubator to mate for three days instead of two. Pairs were still checked twice daily for whether they were engaged in copulation or not (7 checks total, with the 7th check being the morning when the pairs were separated). Males were euthanized following this and females left to lay eggs for 7 days (as opposed to 5 days in Experiment 1). We then euthanized the females, counted the number of eggs laid, and returned tubs to the incubator for a further 7 days to hatch. Following this, any nymphs present were counted. We returned nymphs to the incubator to raise until adulthood, however, these data were not used as the rates of hybridization were too low for any further analysis to be carried out.

### 2.4 Analysis

All statistical analyses were carried out using R statistical software (R Core Team, 2019). For Experiment 1, we treated female and male species + genotype combination as separate factors and looked to see whether there were any interactions between these factors (e.g., if effect of male genotype depended on female genotype). In addition, we made subsets of the data so to make specific a priori comparisons between (a) conspecific crosses and hetero‐specific crosses, and (b) conspecific same‐color morph crosses and conspecific‐different color morph crosses. For Experiment 2, we simply considered “treatment” to have four separate levels, one for each of the experimental crosses. We used generalized linear models (GLMs) with a binomial distribution and logit‐link function to test if there was an effect of treatment on (a) the likelihood of a pair being observed in copula, (b) the likelihood of a female laying eggs, and (c) the likelihood of having nymphs. Using GLMs with an “F” test statistic we investigated whether there was an interaction between male and female genotype on (a) the likelihood of a pair being observed in copula, (b) the likelihood of a female laying eggs, and (c) the likelihood of having nymphs. We also used binomial GLMs to see if there was a relationship between the number of times observed in copula and the likelihood of having offspring. We used a GLM with a quasibinomial distribution to test the effect of conspecific cross on hatching rates in Experiment 1. We used GLMs with a Gaussian distribution to test the effect of treatment (i.e., experimental cross) on (a) the number of eggs laid and (b) the number of nymphs produced. Finally, we used a GLM to investigate whether there was a relationship between the number of times observed in copula and (a) the number of eggs laid and (b) the number of nymphs a pair produced.

## 3 RESULTS

### 3.1 Experiment 1

**Figure 1:**
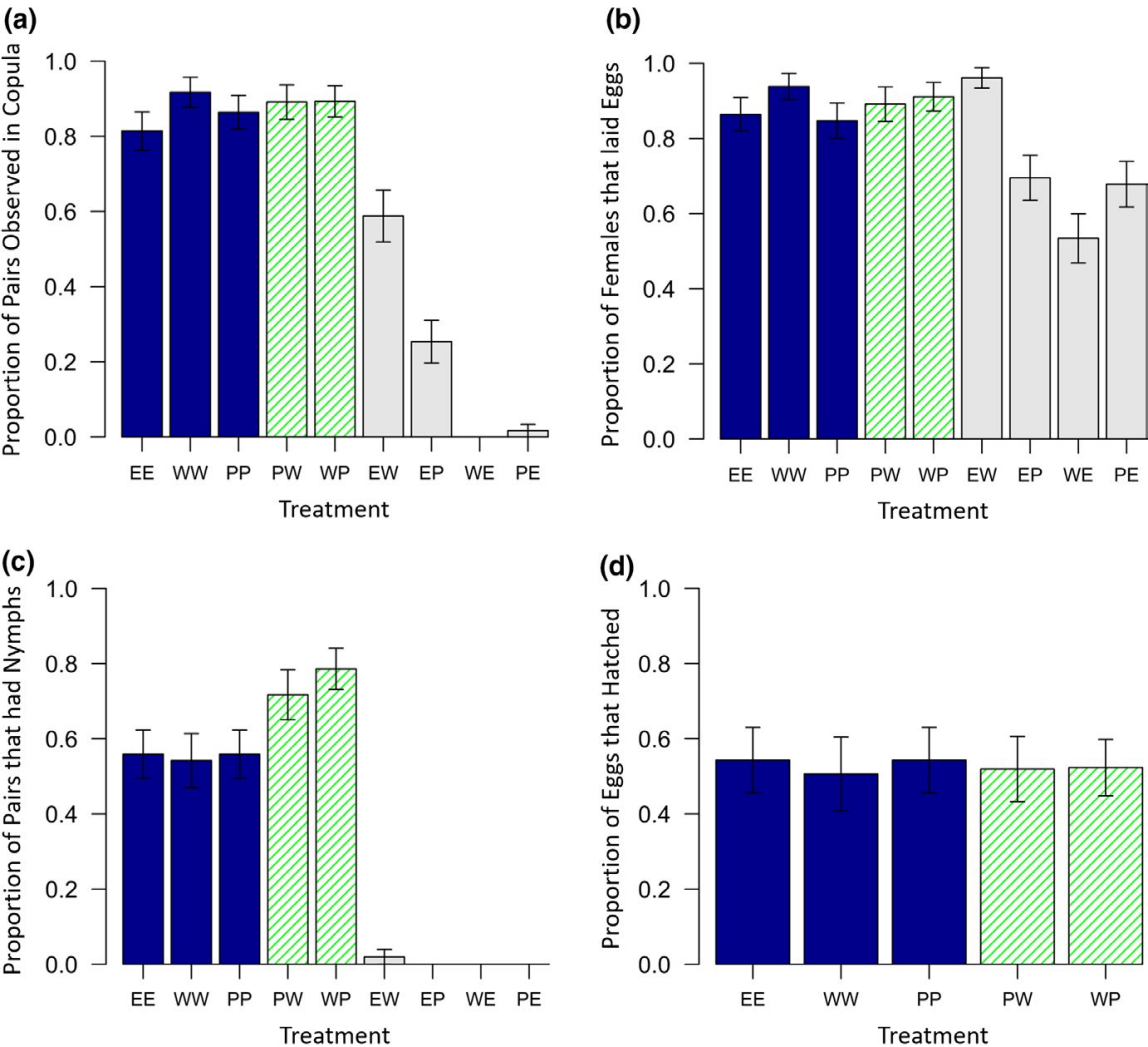
The proportion of pairs which (a) were observed in copula, (b) laid eggs, (c) produced nymphs, and (d) the proportion of eggs that hatched (for conspecific pairs which produced nymphs), depending on treatment, in Experiment 1. Solid blue bars represent conspecific pairs where both individuals were of the same‐color morph, hatched green bars represent conspecific pairs where each individual was of a different color morph, and solid gray bars represent hetero‐specific pairings. Error bars show the standard error. The first letter in each treatment code represents the female species and genotype, the second letter the male species and genotype. E = *L. equestris*, W = wild‐type *L. simulans*, P = pale mutant *L. simulans*

There was a significant main effect of male genotype (*F*_2,486_ = 55.79, *p* < .001) and also a significant interaction between male and female genotype which influenced whether pairs were observed in copula or not (Interaction: *F*_4,486_ = 63.95, *p* < .001; Figure 1a). However, there was no main effect of female genotype (*F*_2,486_ = 0.29, *p* = .75). Unsurprisingly, hetero‐specific pairs were far less likely to engage in copulation than conspecific pairs (χ_1_^2^ = 244.93, *p* < .001). Wild‐type female *L. simulans* were never observed in copula with male *L. equestris*; however, one pale *L. simulans* female was observed copulating with a male *L. equestris* on two separate mating checks. For male *L. simulans* and female *L. equestris* crosses, wild‐type males were twice as likely to be observed in copula (58.8%) than pale males (25.4%; χ_1_^2^ = 12.83, *p* < .001). Within the conspecific treatments, there was no difference in mating rates between pairs of bugs of the same‐color morph (EE, WW, PP) compared to pairs of bugs of different color morphs (PW, WP; χ_1_^2^ = 0.55, *p* = .46).

**Figure 2:**
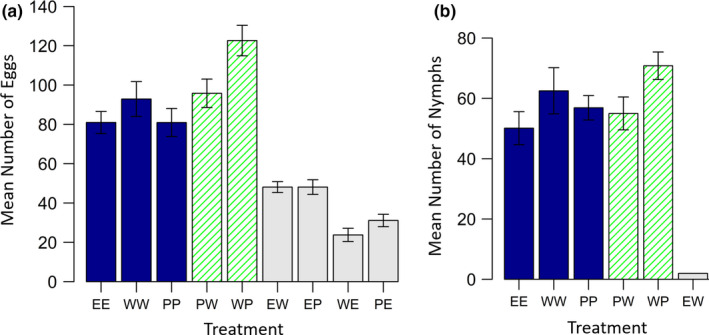
The mean number of (a) eggs produced by each female that laid eggs and, (b) nymphs produced by pairs which had nymphs, depending on treatment, in Experiment 1. Solid blue bars represent conspecific pairs where both individuals were of the same‐color morph, hatched green bars represent conspecific pairs where each individual was of a different color morph, and solid gray bars represent hetero‐specific pairings. Error bars show the standard error. The first letter in each treatment code represents the female species and genotype, the second letter the male species and genotype. Abbreviations as per Figure 1

Females from all treatments laid eggs, and the likelihood of laying eggs also depended on the genotype of the male in the pair (*F*_2,486_ = 15.23, *p* < .001) but not the female's genotype (*F*_2,486_ = 0.69, *p* = .504), although again there was a significant interaction between male and female genotype (Interaction: *F*_4,486_ = 6.32, *p* < .001; Figure 1b). In particular, females from conspecific pairings were more likely to lay eggs than females from hetero‐specific pairings (χ_1_^2^ = 25.39, *p* < .001). Again, there was no difference in the likelihood of laying eggs for females paired with a conspecific male of the same‐color morph, or of a conspecific male of a different color morph (χ_1_^2^ = 0.33, *p* = .57). Conspecific pairings resulted in a higher number of eggs being laid than hetero‐specific pairings when considering only females that laid eggs (*F*_1,397_ = 158.00, *p* < .001; Figure 2a). Within the conspecific treatments, crosses between bugs of different color morphs resulted in a higher number of eggs (*F*_1,236_ = 14.53, *p* < .001), though this was mostly driven by females in the treatment WP laying more eggs. This could possibly because wild‐type females are more fecund than pale females (for example, see Balfour et al., 2018 in which pale *L. simulans* crosses tended to result in fewer eggs than wild‐type crosses). Wild‐type *L. simulans* females (WW) laid more eggs than pale *L. simulans* females (PP) or *L. equestris* (EE) females (Figure 2a).

In terms of nymph production, whether pairs produced nymphs or not depended both on male (*F*_2,486_ = 25.45, *p* < .001) and female (*F*_2,486_ = 20.21, *p* < .001) genotype and there was also a significant interaction between these (*F*_4,486_ = 86.84, *p* < .001; Figure 1c). Only one hetero‐specific pairing resulted in offspring production. A female *L. equestris* and wild‐type *L. simulans* male had two wild‐type hybrid nymphs. Conspecific pairings involving bugs of two different color morphs were more likely to have offspring than conspecific pairings involving two bugs of the same‐color morph (χ^2^_1_ = 11.26, *p* < .001). When considering only pairs which had nymphs, and hence did not experience mating failure, pairs in the treatment WP sired significantly more offspring than pairs in all other conspecific treatments (*F*_1,167_ = 7.68, *p* = .006; Figure 2b). However, for pairs which had nymphs, the hatching rate did not differ between conspecific treatments (χ_4_^2^ = 13.32, *p* = .93; Figure 1d), therefore the higher numbers of nymphs produced in the treatment WP was due to females having laid a greater number of eggs, not due to a higher proportion of eggs hatching.

Finally, there was a positive correlation between the number of times observed in copula and the number of eggs laid (*F*_1,493_ = 259.4, *p* < .001) and the likelihood of having offspring (χ_1_^2^ = 235.41, *p* < .001). Moreover, when considering only pairs which had offspring, pairs observed in copula more often produced a greater number of nymphs (*F*_1,168_ = 6.72, *p* = .01).

### 3.2 Experiment 2

**Figure 3:**
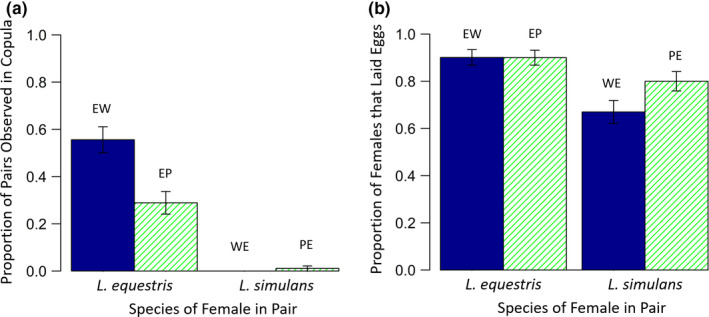
The proportion of pairs which (a) were observed in copula and, (b) laid eggs, depending on treatment, in Experiment 2. Solid blue bars represent hetero‐specific pairs in which both individuals were of the same‐color morph (i.e., the *L. simulans* individual in the pair was wild type), hatched green bars represent hetero‐specific pairs where each individual was of a different color morph (i.e., the *L. simulans* individual in the pair was pale). Error bars show the standard error. Treatment codes are shown above each bar (see main text for explanations)

In the second set of experimental crosses, which only considered hetero‐specific crosses, the likelihood of observing a pair in copula again depended significantly on treatment (χ_3_^2^ = 129.7, *p* < .001; Figure 3a). Wild‐type female *L. simulans* were never observed in copula with *L. equestris* males, and only one pale female *L. simulans* was observed in copula with a male *L. equestris* on three separate mating checks. For pairs involving a female *L. equestris*, pairings involving wild‐type *L. simulans* males were twice as likely to be observed in copula (55.6%) than parings involving a pale male (28.9%; χ_1_^2^ = 12.62, *p* < .001). This is consistent with the results from our first set of crosses (see above).

**Figure 4:**
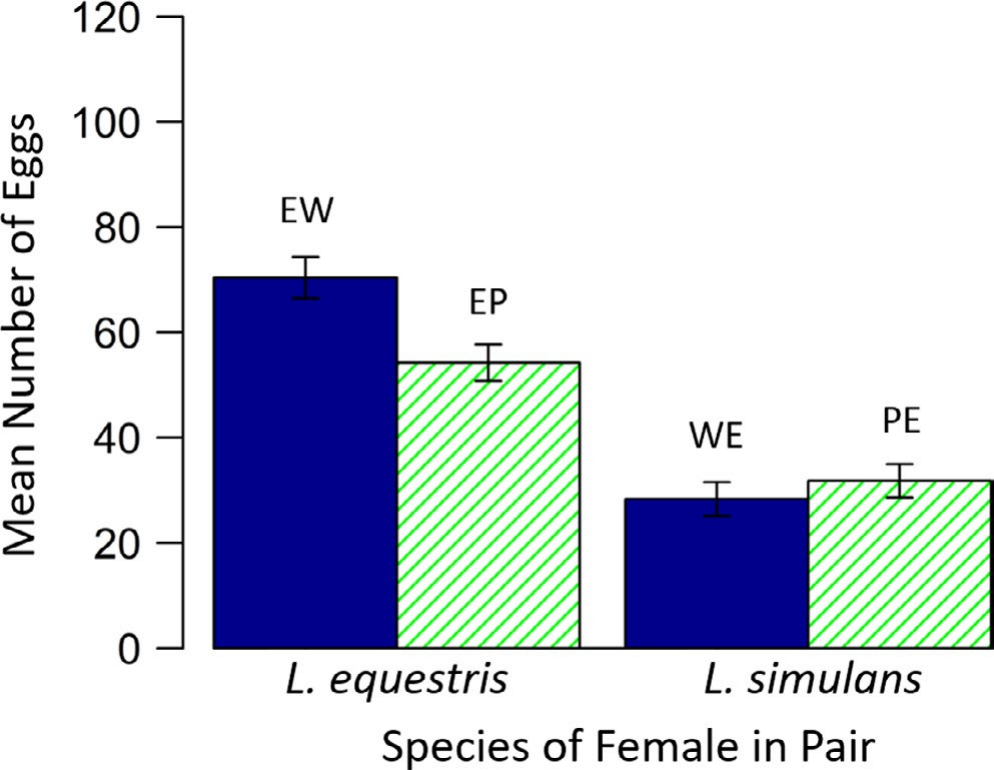
The mean number of eggs laid by females in each pair for females that laid eggs, depending on treatment, in Experiment 2. Solid blue bars represent hetero‐specific pairs in which both individuals were of the same‐color morph (i.e., the *L. simulans* individual in the pair was wild type), hatched green bars represent hetero‐specific pairs where each individual was of a different color morph (i.e., the *L. simulans* individual in the pair was pale). Error bars show the standard error. Treatment codes are shown above each bar (see main text for explanations)

There was a significant effect of treatment on whether pairs laid eggs or not (χ_3_^2^ = 22.54, *p* < .001; Figure 3b). Female *L. equestris* were more likely to lay eggs than female *L. simulans* (χ_1_^2^ = 17.77, *p* < .001) and pairings with wild‐type *L. simulans* tended to be more likely to result in egg production than pairings involving pale *L. simulans*, although the result was marginally nonsignificant (χ_1_^2^ = 3.51, *p* = .061). For females which laid eggs, the number of eggs laid also depended on treatment (*F*_3,288_ = 31.28, *p* < .001; Figure 4), with *L. equestris* females laying more eggs than *L. simulans* females (*F*_1,290_ = 79.4, *p* < .001) but there was only a marginal difference in the number of eggs laid between treatments involving wild‐type and pale *L. simulans* (*F*_1,288_ = 3.72, *p* = .054). Generally, this was driven by females in the treatment EW laying more eggs.

In terms of nymph production, the likelihood of producing offspring differed between treatments (χ_3_^2^ = 11.12, *p* = .01). Only nine of the hetero‐specific pairings (2.5%) resulted in offspring production. Six of these were from the treatment EW, two from the treatment EP and one from the treatment PE (Table 1). This is the first time that hybrids have been obtained from a cross between a *L. simulans* female and a male *L. equestris*. To check that this was genuine and that there had not been an accidental mix‐up with the bugs, the male from this pair was frozen at −18°C and later dissected to confirm the clasper morphology which differs between the two species (Deckert, 1985). The individual was confirmed to be *L. equestris*. Interestingly, this pairing produced more nymphs than any other pairing (Table 1). In concordance with previous findings (Evans et al., 2015; Balfour et al. in review), no hybrids were produced between female wild‐type *L. simulans* and male *L. equestris*. Hybrid nymphs from the three treatments that did result in offspring production were all wild type in color. No pale mutant hybrids were ever observed.

**Table 1:** Details of the pairs which produced hybrid offspring in Experiment 2, including the treatment, the number of eggs laid, the number of hybrid nymphs produced and the proportion of eggs that hatched

Pair ID	Treatment	No. Eggs	No. Nymphs	Proportion of Eggs that Hatched
5	EP	99	1	0.01
67	EW	69	32	0.46
184	EP	134	12	0.09
211	EW	13	1	0.08
215	EW	119	3	0.03
222	EW	100	1	0.01
352	EW	70	3	0.04
358	EW	70	1	0.01
367	PE	185	77	0.42

## 4 DISCUSSION

Our results show that the pale mutation in *Lygaeus simulans* influences prezygotic isolation between *L*. *simulans* and its sister species *L*. *equestris*. First, pale male *L*. *simulans* were less likely to mate with female *L*. *equestris* than wild‐type *L. simulans* males. Second, we did obtain, for the first time, a mating between a female *L. simulans* and a male *L. equestris*, when the female in the pair was the pale color morph. While this latter result was from only two crosses, together our data suggest that the pale mutant may shape likelihood of mating with a hetero‐specific in both male and female *L*. *simulans*. Unfortunately, hybridization was too infrequent to obtain a sample size large enough to compare hybrid fitness between the different experimental crosses. However, one of the female *L. simulans* x male *L. equestris* hybrid matings did produce offspring, again a first. Moreover, within *L*. *simulans*, we once again found evidence for fitness benefits to heterozygotes at the pale color locus (Balfour et al., 2018), with conspecific pairings between pale and wild‐type *L. simulans* having a greater probability of producing offspring than other conspecific pairings, while pairings between wild‐type females and pale males (WP) resulted in a higher number of eggs laid than all other conspecific crosses. Pairs from the treatment WP also had more nymphs, but this was due to having laid more eggs as the hatching success was constant across all conspecific treatments (50.6%–54.3%).

While conspecific pairs were more likely to be observed copulating (87.3%) than hetero‐specific pairs (20.1%), the number of hetero‐specific matings we recorded was nontrivial. Across both experiments, 56.8% of pairs involving a female *L. equestris* and a male wild‐type *L. simulans* were observed in copula at least once. Roughly half as many pairs (27.5%) were observed in copula for female *L. equestris* by male wild‐type *L. simulans* crosses. As the copulation frequency between all conspecific combinations of *L. simulans* pairings did not differ, it seems likely that the lower mating frequency observed in the cross EP compared to EW is not due to an impairment of the pale mutant. What might these data tell us then about the pale color locus and its role in species discrimination?

On the one hand, pale males may be more discriminating than wild types, being less willing to attempt to mate with female *L*. *equestris*. On the other hand, given the color differences, this could be evidence for assortative mating, whereby *L. equestris* females prefer males of the same color as themselves and male *L. equestris*. Within *L*. *simulans*, we have previously shown that pale females are more likely to engage in copulation with pale males than wild‐type males (Balfour, Black, & Shuker, 2020), again potentially hinting toward assortative mating, though we do not yet know if this is due to body color itself, or a difference in cuticular hydrocarbons (CHCs) between pale and wild‐type *L. simulans* (there is preliminary evidence for such CHC differences between morphs: Shuker & Balfour, unpublished data). We also are not sure which sex—or indeed if both sexes—determine pairing and subsequent copulation success. Both males and females could potentially shape any patterns of assortative mating, and we have evidence for both nonrandom mating in terms of both male and female body size in both species (Aumont, Balfour, & Shuker, in prep; Dougherty & Shuker, 2014, 2016).

Evidence that wild‐type female *L. simulans* mate with male *L. equestris* has thus far been rare (Evans et al., 2015 observed one pair of this hetero‐specific cross in copula, though no hybrids were produced). However, as highlighted above, two crosses involving a pale *L. simulans* female and a *L. equestris* male were observed in copula across multiple mating checks in Experiment 2 (where more interspecific crosses were performed c.f. Experiment 1), and one of these pairs produced hybrid offspring. Hybrid offspring were also obtained from the hetero‐specific crosses involving a *L. equestris* female, and all the hybrid offspring were wild type in color. As such, it would be tempting to suggest that pale locus might influence hybrid fitness. However, hybridization rates were extremely low in the present study, with only 10 pairs out of 587 (1.7%) resulting in hybrid offspring. This is much lower than previous hybridization rates (11.6% of hetero‐specific crosses in Evans et al., 2015; 10.1% of female *L. equestris* and male *L. simulans* crosses in Balfour et al. unpublished data).

Because so few hybrids were produced, we did not have enough data to compare the fitness between hybrids of different hetero‐specific crosses to see if there was variation in the fitness of hybrids from different crosses and with different genetic backgrounds. Therefore, we cannot assess whether the pale color locus could influence postzygotic reproductive isolation. We do wish to note here, however, that the genotype of the male *L*. *simulans* (pale mutant vs. wild type) does not seem to influence the number of eggs laid by *L. equestris* females mated to them. For these females which laid eggs, there was no difference in the number of eggs laid between treatments EW and EP in Experiment 1 (note that not all females that laid eggs were observed mating, and unmated females will lay unfertilized eggs in this species) and the differences in Experiment 2 are likely due to the higher mating frequencies in the cross EW, as there is evidence that oviposition frequency increases with copulation (Sillén‐Tullberg, 1984). More data are clearly needed to tease apart pre‐ and postzygotic effects though, including sperm transfer and use.

The highly pleiotropic nature of the locus (Balfour et al., 2018) suggests that the causative mutation is either in a gene or the regulatory sequence of a gene that is highly pleiotropic (perhaps involved in many developmental and/or signaling pathways), or that color patterns in *Lygaeus* are instead controlled by a supergene complex, and that the pale mutant is a mutation that has arisen among a larger co‐segregating region, as would be the case with a polymorphic inversion system (Black & Shuker, 2019; Charlesworth, 2015; Thompson & Jiggins, 2014). While our discussion here for the moment is admittedly speculative, these data add to the list of behaviors that are influenced by this locus. Moreover, even though the mutation we have studied here arose in the laboratory, a potentially rather similar “gray” mutant has been observed in cultures of the sibling species *L*. *equestris* originally collected in northern Italy (Sillén‐Tullberg, 1985). While only suggestive at best, this older record hints that there may be segregating variation in natural populations of *L*. *simulans* and *L*. *equestris* at this, or another closely related, color locus. The variation we have seen across studies in hybridization success also suggests that there may be segregating variation in our laboratory populations for postzygotic genetic incompatibilities, variation that has been seen in natural populations of other species (e.g., the grasshopper *Chorthippus parallelus*: Shuker et al., 2005). As such, this sibling‐species pair perhaps provides a new opportunity for scrutinizing the genetic basis of reproductive isolation, both in terms of the observed differences in prezygotic isolation associated with the pale locus reported here, and in terms of variation in postzygotic isolation.

## CONFLICT OF INTEREST

The authors have no competing interests.

## AUTHOR CONTRIBUTION

**Vicki L Balfour:** Conceptualization (equal); Data curation (equal); Formal analysis (lead); Investigation (lead); Methodology (equal); Supervision (equal); Visualization (equal); Writing‐original draft (equal); Writing‐review & editing (equal). **Daniella Black:** Data curation (equal); Formal analysis (supporting); Investigation (supporting); Writing‐review & editing (supporting). **David M. Shuker:** Conceptualization (equal); Formal analysis (supporting); Investigation (supporting); Methodology (equal); Supervision (equal); Writing‐original draft (equal); Writing‐review & editing (equal).

## Data Availability

Our data have been archived in Dryad. (https://doi.org/10.5061/dryad.qbzkh18fz).
